# Emotional Intelligence, Emotional Regulation Strategies, and Subjective Well-Being Among University Teachers: A Moderated Mediation Analysis

**DOI:** 10.3389/fpsyg.2021.811260

**Published:** 2022-01-10

**Authors:** Jingrong Sha, Tianqi Tang, Hong Shu, Kejian He, Sha Shen

**Affiliations:** ^1^College of Educational Science and Technology, Northwest Minzu University, Lanzhou, China; ^2^Key Laboratory of China’s Ethnic Languages and Information Technology of Ministry of Education, Northwest Minzu University, Lanzhou, China; ^3^Faculty of Law and Public Administration, College of Science and Technology, Guizhou University, Guiyang, China

**Keywords:** emotional intelligence, emotional regulation strategies, subjective well-being, university teachers, effort-reward imbalance

## Abstract

This study aimed to explore the mediating role of emotional regulation strategies in the relationship between emotional intelligence (EI) and subjective well-being (SWB) among Chinese university teachers, and evaluate whether effort-reward imbalance moderated the mediating effect of emotional regulation strategies. A total of 308 Chinese university teachers were recruited for this study. The results showed that emotional regulation strategies played a partial mediating role in the relationship between EI and SWB. Moreover, an effort-reward imbalance moderated the relationship between emotional regulation strategies and SWB. For individuals with more balanced perceptions, EI had a significant effect on SWB *via* cognitive reappraisal, while for individuals with more imbalanced perceptions, EI did not have a significant effect on SWB *via* cognitive reappraisal. These findings provide a better understanding of the effects of EI and emotional regulation strategies on SWB, which could provide interventions for promoting SWB among teachers.

## Introduction

Teachers at universities experience high levels of stress and job burnout owing to the challenging nature of their work (Teles et al., 2020), thus they have a significantly lower level of subjective well-being (SWB) than other professionals (Grenville-Cleave and Boniwell, 2012). SWB is regarded as “evaluation of life satisfaction and positive and negative affective reactions to one’s life” (Busseri and Sadava, 2011; Chan, 2013), which is believed to play an important role in physical and mental health (Pressman and Cohen, 2005; Tsaousis et al., 2007). Many studies have shown that SWB is important for teachers. It is not only associated with their professional performance (Rahm and Heise, 2019) and mental health (Xin et al., 2021), but also influences students’ cognitive outcomes (Pietarinen et al., 2014; Hung et al., 2016) and well-being (Xin et al., 2021).

Considering the importance of SWB, a great deal of research attention has been devoted to this field, with particular attention to identifying the factors and mechanisms that affect it (Koydemir and Schütz, 2012). Teachers can be regarded as emotional workers who need to be sensitive to their jobs (Yin, 2015), and there is no doubt that emotional intelligence (EI) is an important factor that influences SWB. EI is defined as the ability to evaluate and express emotions, regulate emotions, and use emotional content in thinking and action (Mayer and Salovey, 1993). Previous studies have shown that one of the most reliable correlates of EI is SWB (Sánchez-Álvarez et al., 2016) and EI has been confirmed to be a strong predictor of SWB (Vergara et al., 2015; Lin et al., 2016). For example, Sánchez-Álvarez et al. (2016) conducted a meta-analysis of 25 studies with 8,520 participants, and the results showed that EI was positively correlated with SWB. Huang et al. (2018) used a structural equation model to conduct an empirical test on the survey data of 412 university students from two universities in South China and found that people with higher EI reported higher levels of SWB. In addition, studies have found that the improvement of teachers’ EI can enhance their SWB (Vesely et al., 2013).

Although previous studies have indicated that EI is closely related to SWB, the relationship between EI and teachers’ SWB is inconsistent (Lee et al., 2019). For example, Hassan (2019) explored the relationships between EI and SWB among university teachers and found a positive correlation between EI and SWB. However, some studies have found that EI has little influence on SWB. For example, Zeidner and Olnick-Shemesh (2010) found that EI had limitations in predicting SWB.

A possible explanation is that the relationship between EI and teachers’ SWB is mediated or moderated by other factors. For example, previous studies have indicated that the relationships between EI and SWB may be influenced by emotion regulation strategies. Emotional regulation strategies include cognitive reappraisal and suppression of expression (Gross and John, 1998). Cognitive reappraisal is a form of cognitive change that involves construing a potentially emotion-eliciting situation in a way that changes its emotional impact (Lazarus and Alfert, 1964). Expressive suppression is a form of response regulation modulation that involves the suppression of persistent emotional expression behavior (Gross and John, 1998). Previous studies have shown a significant relationship between EI and emotional regulation strategies. For example, Quintana-Orts et al. (2020) confirmed there is a positive association between EI and emotion regulation strategies. Specifically, people with higher EI are more likely to choose effective emotional regulation strategies (Śmieja-Nęcka et al., 2011). In addition, studies have shown a significant relationship between emotion regulation strategies and SWB. For instance, Katana et al. (2019) discussed the influences of emotion regulation strategies on SWB, and the results showed that cognitive reappraisal aimed at increasing positive emotions was positively associated with higher SWB; suppression of positive emotion expression was negatively correlated with SWB. Therefore, do emotion regulation strategies mediate the relationships between EI and SWB among university teachers?

In addition, the relationship between EI, emotion regulation strategies, and SWB may be moderated by an effort-reward imbalance. Effort-reward imbalance is a source of work-related stress (Siegrist, 2010). According to affective events theory (Weiss and Cropanzano, 1996), the emotional experience accumulated in the work environment, together with other factors (including personality), shapes employees’ work attitudes. In addition, affective event theory (Weiss and Cropanzano, 1996) proposed that the emotional state at work is a key carrier of the influence of personality and organization on job satisfaction and performance. Previous studies indicated that individuals with more positive affects at work are more likely to have higher EI (Lopes et al., 2006; Kafetsios and Zampetakis, 2008) and are prone to use more positive emotion regulation (Mérida-López and Extremera, 2017). Thus, effort-reward imbalance may be a potential moderator for the mediating effect of emotion regulation strategies between EI and SWB.

Based on previous studies and related literature, this study aimed to investigate the mediating role of emotional regulation strategies between EI and SWB and explored whether effort-reward imbalance moderated the mediating effect of emotional regulation strategies between EI and SWB among Chinese university teachers. Our hypotheses were as follows: (1) EI and emotional regulation strategies had positive effects on SWB; (2) emotional regulation strategies played a mediating role in the relationship between EI and SWB; and (3) effort-reward imbalance moderated the mediating effect of emotional regulation strategies between EI and SWB.

## Materials and Methods

### Participants

We distributed 350 questionnaires to three universities in western China. The teachers ranged in age from 23 to 58 years, with a mean age of 38.4 (SD = 9.97). After excluding incomplete questionnaires, 308 participants’ responses were used in this study. After completing the questionnaire, all participants received a gift as a reward. Please see Table 1.

**TABLE 1:** Socio-demographic characteristics of the participants (*n* = 308).

		Groups	Frequency (%)
Gender	Female	142	46.1%
	Male	166	53.9%
Age	≤35	170	55.2%
	36–50	106	34.5%
	≥50	32	10.3%
Family background	Rural	55	17.9%
	Towns	253	82.1%
Only-child	Yes	132	42.9%
	No	176	57.1%

#### Emotion Regulation Questionnaire

This study used an emotion regulation questionnaire (ERQ) based on the two-stage emotional regulation process model proposed by Gross (2003). A total of 10 items were divided into two dimensions: cognitive reappraisal (six items) and expression suppression (four items). Each item was answered on a seven-point scale (1 = very inconsistent, 7 = very consistent). In this study, the Cronbach’s alpha coefficient of the cognitive reappraisal score was 0.85, and the Cronbach’s alpha coefficient of the expression suppression score was 0.68.

#### Emotional Intelligence Scale

Emotional intelligence was measured using the Emotional Intelligence Scale (EIS) developed by Law et al. (2004). It contains 16 items, each item is answered on a seven-point scale (1 = strongly disagree, 5 = strongly agree), and the total sum score based on the 16 items varies between 16 and 112. The higher the score that participants receive, the higher their EI. The Cronbach’s alpha coefficient in this study was 0.94.

#### Effort-Reward Imbalance Questionnaire for Teachers

This study adopted the EFR questionnaire developed by Ren et al. (2019), which consists of two components (effort and reward). Effort contains 14 items, and the reward contains 14 items. Each item was answered on a five-point scale (1 = strongly disagree, 5 = strongly agree). According to Siegrist et al. (2004), the effort-reward ratio was computed for every respondent according to the formula e/(r × c) where “e” is the sum score of the effort scale, “r” is the sum score of the reward scale and “c” defines a correction factor for different numbers of items in the nominator and denominator, and the correlation factor of this study’s questionnaire is 1 (14/14). The higher the ratio score participants get, the greater the imbalanced participants have, and the lower the ratio score participants get, the more balance participants have. The Cronbach’s alpha coefficient of effort and reward in this study was 0.94 and 0.92, respectively.

#### Subjective Well-Being Scale

The study used the Chinese version of the SWB Scale (Duan, 1996), which consists of 18 items. Each item was answered on a five-point scale (1 = strongly disagree, 5 = strongly agree). The higher the total score, the more subjective the well-being of the participants. Cronbach’s alpha coefficient in this study was 0.85.

### Procedure

Data were collected using online questionnaires. First, we contacted the dean of academic affairs of the university, stated the purpose of the study, and received approval. Teachers were then invited to complete the questionnaire independently on weekdays.

### Ethics

This study was approved by the Northwest Ethics Committee. Before participating in this study, all participants provided written informed consent to participate in the study.

### Analytical Strategy

SPSS software (version 25.0) was used for data analysis. We first established the relationships among EI, effort-reward imbalance, emotional regulation strategies (cognitive reappraisal and expressive suppression), and SWB. Then, we used the SPSS macro PROCESS program to evaluate the mediating effect of emotional regulation strategies on EI and SWB and the moderating effect of effort-reward imbalance.

## Results

### Correlation Analysis of Emotional Intelligence, Emotional Regulation Strategies, Effort-Reward Imbalance, and Subjective Well-Being

Pair correlation tests were conducted for each variable first, and the correlation coefficients among EI, emotional regulation strategies, effort-reward imbalance, and SWB are shown in Table 2.

**TABLE 2:** Statistical results of correlation analysis of main variables (*n* = 308).

Variable	*M* (SD)	1	2	3	4	5
EI	62.88(14.41)	1	0.34**	−0.26**	−0.16**	0.40**
Cognitive reappraisal	34.01(6.27)		1	0.05	0.01	0.35**
Expressive suppression	16.15(5.79)			1	0.10	−0.17**
Effort-reward imbalance	1.16(0.60)				1	−0.20**
SWB	78.47(9.01)					1

**p < <0.01.

### The Relationships Between Emotional Intelligence, Emotional Regulation Strategies, and Subjective Well-Being

**FIGURE 1:**
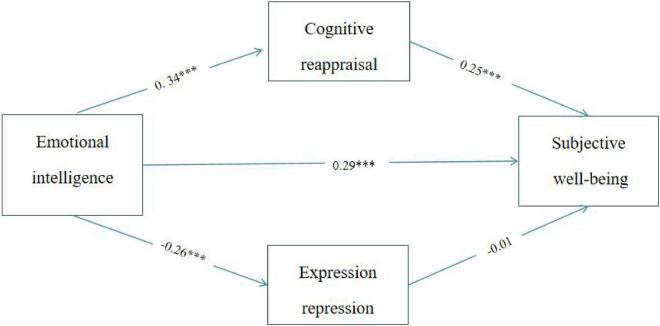
The relationship models of EI, emotional regulation strategies, and SWB. ***p < 0.001.

To analyze the influence of EI on SWB and the role of emotional regulation strategies, the SPSS macro PROCESS program was used to evaluate the mediating effect. Two regression equations were used to evaluate the following: first, the direct effect of EI on SWB; second, the mediating effects of cognitive reappraisal and expressive suppression between EI and SWB. The regression analysis results are listed in Table 3. The results showed that EI significantly predicted SWB (β = 0.39, *t* = 7.59, *p* = 0.000), cognitive reappraisal (β = 0.34, *t* = 6.37, *p* = 0.000), and expression suppression (β = −0.26, *t* = −4.65, *p* = 0.000). Also cognitive reappraisal significantly predicted SWB (β = 0.25, *t* = 4.63, *p* = 0.000), but expression suppression did not significantly predict SWB (β = −0.10, *t* = −1.93, *p* = 0.05). As shown in Figure 1, there was a mediating effect of cognitive reappraisal on the effect of EI on SWB.

**TABLE 3:** Mediation effect model test (*n* = 308).

Variable	Model 1			Model 2		
	SWB			SWB		
	Effect of value	SE	*t*	Effect of value	SE	*t*
EI	0.39	0.03	7.59***	0.29	0.04	5.06***
Cognitive reappraisal				0.25	0.08	4.63***
Expression suppression				–0.10	0.08	–1.93
*R* ^2^	0.15			0.22		
*F*	57.72**			28.26***		

**p < 0.01; ***p < 0.001.

Bias correction non-parametric percentage Bootstrap was further used to test the mediating effect, and the results are shown in Table 4. The direct effect value of EI on SWB was 0.17, accounting for 70.83% of the total effect, and the 95% interval was (0.10, 0.24), indicating that the direct effect was significant. The indirect effect value of cognitive reappraisal on EI and SWB was 0.07, accounting for 29.17% of the total effect, with a 95% interval of (0.02, 0.13), indicating a significant mediating effect.

**TABLE 4:** Bootstrap analysis of significance test of mediation effect (*n* = 308).

The path	Effect of value	Effect of the amount	Bootstrap 95% confidence interval down line	Bootstrap 95% confidence interval upper line
Direct effect (A→C)	0.17	85.71%	0.10	0.24
Indirect effect (A→B→C)	0.07	14.29%	0.02	0.13
Total effect	0.24	100%	0.18	0.31

*A, EI; B, Cognitive Reappraisal; C, SWB.*

### The Moderated Mediation Analysis of Effort-Reward Imbalance

Table 5 shows the results of the moderated mediation analysis of effort-reward imbalance. In Model 59 (Table 5), the interaction term between EI and effort-reward imbalance was not significantly associated with cognitive reappraisal (β = −0.06, *p* = 0.06) and SWB (β = 0.07, *p* = 0.16), indicating that effort-reward imbalance did not moderate the relationship between EI and cognitive reappraisal, as well as the relationship between EI and health SWB. Moreover, the interaction term between cognitive reappraisal and effort-reward imbalance was significantly related to SWB (β = −0.35, *p* = 0.01), which showed that the effort-reward imbalance moderated the relationship between cognitive reappraisal and SWB.

**TABLE 5:** Results of the moderated mediation analysis.

Independent variables	Model 59		Model 14	
	Subjective well-being		Subjective well-being	
	Coefficient	*t*	Coefficient	*t*
Emotional intelligence	0.17	5.18***	0.17	5.16***
Cognitive reappraisal	0.33	4.25***	0.32	4.19***
Effort-reward imbalance	–2.33	−3.08**	–2.22	−2.95**
Emotional intelligence × effort-reward imbalance	0.07	1.40		
Cognitive reappraisal × effort-reward imbalance	–0.34	−2.44*	–0.27	−2.08*
*R* ^2^	0.24		0.24	
*F*	19.80***		24.18***	

*p < 0.05; **p < 0.01; ***p < 0.001.

To further examine the moderation mediation effect, we conducted Model 14 (Table 5).

**FIGURE 2:**
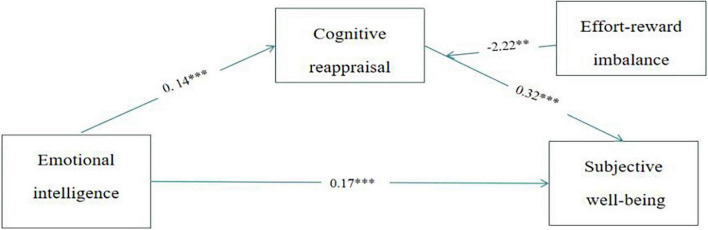
The moderation mediate effect. **p < 0.01; ***p < 0.001.

The interaction term between cognitive reappraisal and effort-reward imbalance was significantly associated with SWB (β = −0.28, *p* = 0.04), as shown in Figure 2, the effort-reward imbalance moderated the effect of cognitive reappraisal on SWB. The index of moderated mediation was significant (β = −0.04, 95% CI = −0.083, −0.005). For individuals with more balanced perceptions, EI had a significant effect on SWB *via* cognitive reappraisal (β = 0.07, 95% CI = 0.039, 0.117). As for individuals with more imbalanced perceptions, EI had no significant effect on SWB *via* cognitive reappraisal (β = 0.02, 95% CI = −0.013, 0.065).

## Discussion

This study evaluated the relationships between EI, emotional regulation strategies, effort-reward imbalance, and SWB among Chinese university teachers. The results showed that EI and emotional regulation strategies were significant predictors of SWB. In addition, this study also revealed that EI affects SWB through emotional regulation strategies. Moreover, an effort-reward imbalance moderated the relationship between emotional regulation strategies and SWB.

The results of this study show that EI positively predicted SWB, which was consistent with previous studies (e.g., Sánchez-Álvarez et al., 2016; Szczygieł and Mikolajczak, 2017). According to EI theory, as individuals with high EI are good at understanding and managing their emotions, they are more likely to cope better with the stresses and troubles of daily life (Zeidner et al., 2012). In addition, people with high EI are more likely to have good interpersonal relationships and prosocial behavior (Wood et al., 2007). Therefore, people with high EI are prone to have a higher level of SWB (Zeidner et al., 2009).

In addition, the results of this study indicate that emotional regulation strategies play a mediating role in the relationship between EI and SWB. Specifically, this study indicated that only cognitive reappraisal mediated the relationship between EI and SWB among Chinese university teachers. Previous studies have suggested that cognitive reappraisal and expression suppression are different aspects and have different neural mechanisms (Gross and John, 2003). Cognitive reappraisal exhibits preferential regulatory advantage, which occurs at an early stage of emotion generation. Therefore, the expression of emotion can change before the emotional response is fully generated (Schutte et al., 2009). In addition, instead of avoiding affective states, cognitive reappraisal deals with emotions by living with negative emotions. Specifically, in an embarrassing situation, they do their best to alter the circumstances and change the emotional consequences of the situation (Schutte et al., 2009). Moreover, studies have shown that cognitive reassessment not only reduces negative emotions and behavioral expression, but also requires relatively few cognitive resources, which can be used effectively in the social environment (Gross, 1999; Richards and Gross, 2000; John and Gross, 2004; Gross et al., 2006). Therefore, cognitive reevaluation, as a healthy emotion regulation strategy, can better promote an individual’s SWB (Haga et al., 2009).

More importantly, the study found that effort-reward imbalance moderated the relationship between emotional regulation strategies and SWB. For individuals with more balanced perceptions, EI had a significant effect on SWB *via* cognitive reappraisal, while for individuals with more imbalanced perceptions, EI had no significant effect on SWB *via* cognitive reappraisal. These results are in line with previous theories and studies. Affective events theory and affective event theory all proposed that the emotional experience accumulated in the work environment shapes employees’ work attitudes. In addition, previous studies have shown that work affects are associated with EI and emotion regulation. More importantly, the results indicate that, compared with negative affect, the positive affect is stronger. This is an important finding that supports the growing evidence that positive affect takes precedence over negative affect as a predictor of work outcomes (Thoresen et al., 2003). As a source of human power (Isen, 2003) and positive affect promote the construction of personal and social resources (Fredrickson, 2001; Lyubomirsky et al., 2005). Therefore, the individuals with higher EI are more likely to adopt positive emotion regulation strategies which follow these “broadening and build” strategies, leading to higher a level of SWB.

This study had some limitations. First, the study used a cross-sectional design, which made it difficult to explore causal interpretations of these variables. A longitudinal study design is required in the future. Second, participants were recruited from the western cities of China, which may limit the generalization of the findings. Compared with the eastern coastal cities, the economy of northwest China is relatively backward, and previous studies have indicated that the economic level is closely related to SWB (Ngamaba et al., 2018; Ding et al., 2021) and EI (Shukla and Srivastava, 2016; Karapetyan, 2021). Future studies should expand the city of the participants.

These results provide a better understanding of the relationship between EI, emotional regulation strategies, and SWB, and expand the reports of potential causes of teachers’ SWB from other studies. In addition, the present study has important theoretical significance for developing the theory of SWB, which expands the study of potential factors of university teachers’ SWB and uncovers the mechanisms between EI and SWB. Although many studies have explored the relationships between EI and SWB and tried to explore the potential mechanism between them, only a few studies have investigated the role of emotional regulation strategies. Thus, whether emotional regulation strategies mediate the relationship between EI and SWB remains unclear. This study provides evidence of the mediating role of emotional regulation strategies. More importantly, the study provides evidence for developing interventions to improve teachers’ SWB. For example, educational institutions and government departments can improve teachers’ intelligence through a series of activities, such as group psychological counseling and mental health lectures. In addition, for teachers with low EI, cognitive therapy can be used to improve their emotional regulation strategies to enhance SWB.

## Data Availability Statement

The raw data supporting the conclusions of this article will be made available by the authors, without undue reservation.

## Ethics Statement

This study was reviewed and approved by Ethics Committee of the College of Educational Science and Technology of Northwest Minzu University. Written informed consent was obtained from all participants for their participation in this study.

## Author Contributions

JS proposed the initial idea. TT and HS conducted the research, collected, and analyzed the data. SS has made significant contributions by redesigning and modifying the framework of the present study. All authors participated in writing the manuscript and approved the submitted version.

## Conflict of Interest

The authors declare that the research was conducted in the absence of any commercial or financial relationships that could be construed as a potential conflict of interest.

## Publisher’s Note

All claims expressed in this article are solely those of the authors and do not necessarily represent those of their affiliated organizations, or those of the publisher, the editors and the reviewers. Any product that may be evaluated in this article, or claim that may be made by its manufacturer, is not guaranteed or endorsed by the publisher.
